# Complete Dosimetric Characterization of an In-House Manufactured SFRT Grid Collimator by 3D Printing with PLA-W Composite Filament

**DOI:** 10.3390/polym17111496

**Published:** 2025-05-28

**Authors:** José Velásquez, Melani Fuentealba, Mauricio Santibáñez

**Affiliations:** 1 Departamento de Cs. Físicas, Universidad de La Frontera, Temuco 4811230, Chile; jose.velasquez@ufrontera.cl; 2 Laboratorio de Radiaciones Ionizantes, Universidad de La Frontera, Temuco 4811230, Chile; 3 Programa de Doctorado en Ingeniería, Facultad de Ingeniería y Ciencias, Universidad de La Frontera, Temuco 4811230, Chile

**Keywords:** grid collimator, 3D printing, PLA-W, SFRT, grid dosimetry

## Abstract

This study presents a comprehensive dosimetric characterization and commissioning of a grid-type collimator manufactured via 3D printing using PLA-W composite filament, following an international protocol for small-field dosimetry. PLA doped with high concentrations of tungsten (>90% *w*/*w*) enables the fabrication of miniaturized collimators (<1 cm) with complex geometries, suitable for non-conventional radiotherapy applications. However, accurate assessment of spatial dose modulation is challenged by penumbra overlap between closely spaced beamlets, limiting the application of conventional instrumentation and protocols. To address this, absolute and relative dose distributions were evaluated for various radiation field configurations (number of beamlets) in both lateral and depth directions. Measurements were performed according to the IAEA TRS-483 protocol, using micro-ionization chambers and diode detectors. Additionally, long-term stability assessments were carried out to evaluate both the structural integrity and modulation performance of the printed grid over time. Point dose measurements using the same detectors were repeated after one year, and 2D surface dose distributions measured with EBT3 films were compared to SRS MapCHECK measurements two years later. The generated radiation field size of the central beamlet (FWHM) differed by less than 0.2% (15.8 mm) from the physical projection size (15.6 mm) and the lateral transmission due simultaneous beamlets resulted in FWHM variations of less than 3.8%, confirming manufacturing precision and collimator capability. Output factor measurements increased with the number of beamlets, from 0.75 for a single beamlet to 0.82 for the full beamlets configuration. No significant changes were observed in the depth of maximum dose across the different beamlets configurations (1.20 ± 0.20 cm). On the other hand, the long-term evaluations show no relevant changes in the FWHM or VPR, confirming the performance and reliability of the system. These results support the clinical feasibility and lasting performance stability of in-house manufactured grid collimators using PLA-W filaments and accessible 3D printing technology.

## 1. Introduction

Spatially Fractionated Radiation Therapy (SFRT) is a treatment that involves delivering high doses of radiation in a single fraction to a target volume in a heterogeneous manner. The dose delivery is achieved using individual beams, with the use of accessories whose purpose is to spatially modulate the radiation dose, with examples of such accessories being grid-shaped blocks. Another modality to achieve this effect is through multileaf collimators, where this process is done virtually with the assistance of a treatment planning system in more complex techniques, a process known as LATTICE [1], as well as microbeam-based synchrotron [2,3]. These techniques require a more complex treatment planning process and additional quality control procedures [1]. Originally, this therapy modality had its beginnings in 1909 under the name of “GRID therapy” with a peak in the 1930s [3]. Over time, the use of the grid decreased considerably, being replaced by emerging treatment techniques. However, in recent years, there has been renewed interest due to treatment benefits that are not achieved with modern techniques [1,4].

From its beginning, SFRT has been primarily used for treating bulky malignant tumors in conjunction with high-dose techniques such as stereotactic radiosurgery (SRS) or stereotactic body radiotherapy (SBRT) in a dose range of 10–20 Gy with megavoltage beams. The results obtained have been remarkable, such as symptom relief in patients with pain, tumor regression, increased tumor control due to an increase in Biologically Effective Dose (BED) [5], and minimal toxicity in adjacent organs [3,6,7]. Due to its dose distribution, organs such as the skin and subcutaneous tissue achieve greater tolerance to radiation, allowing much higher doses to be delivered to the tumor while avoiding the toxicity typically produced by conventional treatments [2,8,9,10]. Over time, its application in other pathologies has increased, achieving clinical response rates of over 50% [11,12,13].

High-density block confection dates back to the early days of radiation therapy with the manufacturing of collimators or compensators through subtractive manufacturing of Cerrobend alloys (26.7% lead, 50% bismuth, 13.3% tin, and 10% cadmium) [14]. Several years ago, the production of these blocks began to be addressed through manufacturing via 3D printing, proposing the creation of a negative mold with defined geometries based on dose fluence maps, where the Cerrobend alloy (previously melted) is poured, allowing the production of a solid compensator [15]. However, the main drawback of this alloy is the toxicity of its constituent materials, such as lead and cadmium. In the case of lead, it is classified as extremely hazardous (level 4) by the National Fire Protection Association [16], and cadmium contains toxic, carcinogenic, and stimulant elements [17]; thus, its discontinuation in the clinical environment has been recommended [18].

Over the past few years, the exploration of constructing compensating blocks entirely through additive manufacturing techniques such as 3D printing has been undertaken. This method enables the creation of objects through a layer-by-layer bonding process [19], using the Fused Filament Fabrication (FFF) modality. This method utilizes a continuous filament of thermoplastic material, with Polylactic Acid (PLA) being one of the most used materials in the biomedical field [3,20,21], given its non-toxic, biocompatible, and biodegradable properties [22]. However, PLA has a low capacity for attenuating high-energy photon radiation (MV energies) [23], requiring doping with a high-Z element. The initial clinical studies that explored manufacturing with PLA compounds doped with metals used copper to infuse into the polymer, in concentrations of up to 80%, successfully generating veterinary radiotherapy compensating blocks for 225 kV photon energies [24]. Nonetheless, these energy ranges are not commonly used in human radiotherapy, and such PLA compounds still produce low levels of attenuation for 6 MV or higher photon energies (as used in linear accelerators), necessitating the development of metal-based compounds with higher Z values.

Recently, Velásquez et al. (2024) [25] demonstrated the feasibility of using PLA composite filaments with high tungsten content (>90% *w*/*w*) for the fabrication of grid collimators via 3D printing. Their work focused primarily on the material’s physical properties and attenuation performance, establishing its potential for clinical use as a convenient, rapid, and cost-effective alternative to traditionally machined accessories. Importantly, this approach opens the possibility for in-house production of radiotherapy components, removing dependence on external manufacturing [25,26].

However, although the previous study confirmed the physical viability of PLA-W materials for radiation shielding, it did not address the comprehensive dosimetric behavior of the printed grid under clinical beam conditions, nor its long-term performance in treatment settings. The characterization of spatial dose modulation, critical for safe and effective clinical implementation of SFRT, remains an open question. This includes parameters such as lateral and depth dose profiles, output factors, modulation stability, and the impact of overlapping penumbras in small, closely spaced beamlets.

This study presents a comprehensive experimental dosimetric characterization of a grid collimator manufactured in house using PLA-W and accessible FFF 3D printing technology. Following international guidelines for small-field dosimetry (IAEA TRS-483 [27]), the evaluation employs high-precision instrumentation, including micro-ionization chambers, diode detectors, and 2D array systems, to assess both absolute and relative dose distributions across different radiation field configurations over the grid (multiple beamlets). The long-term stability of the collimator was also evaluated, demonstrating consistent structural integrity and dose modulation performance over two years. These findings support the technical robustness and clinical feasibility of the proposed solution. By addressing the critical gap between material feasibility and clinical implementation, this study validates the use of PLA-W-based, 3D-printed grids for SFRT and provides essential evidence supporting their routine use in radiotherapy centers with in-house fabrication capabilities.

## 2. Materials and Methods

### 2.1. Grid Manufacturing

The design and construction of the grid were conducted using 3D printing through the FFF material extrusion (MEX) technique, based on the design by Velásquez et al. [25]. The manufacturing material used was based on W-doped PLA at 93% *w*/*w* (density 7.51 g/cm^3^)—Rapid 3D Shield model from The Virtual Foundry brand (The Virtual Foundry, Inc. Stoughton, WI, USA) [28]. This compound has widely reported properties such as the biocompatibility of PLA and the low toxicity of tungsten [29], allowing it to meet safety and dosimetric standards for use in clinical accessories.

The printer used was the PRUSA i3 MK3S printer (Prusa Research, Prague, Czech Republic) operating under a direct extrusion system, with the advantage over the Bowden-type printing system of avoiding filament diameter irregularity, making it a suitable option for printing filaments with metallic compounds. The printing parameters used for the elaboration of the grid-type collimator include the use of a 0.6 mm diameter reinforced steel nozzle, 0.45 mm layer height, 100% infill, nozzle temperature of 235 °C, and deposition speed of 52.5 mm/s.

**Figure 1:**
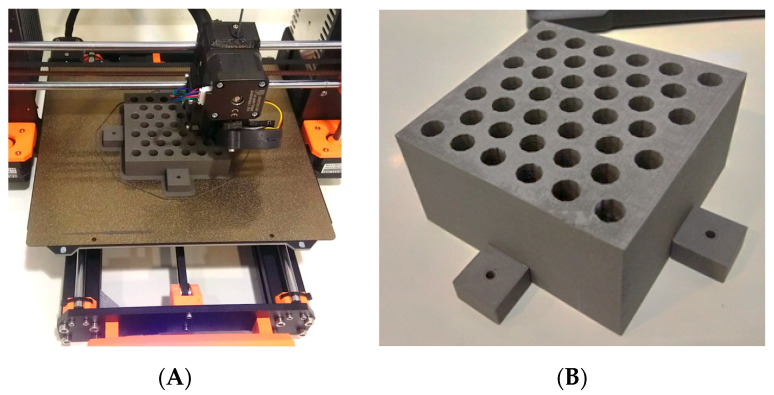
(**A**) shows the 3D printing process using the material extrusion technique (MEX) of the grid collimator with PLA-W filament on a PRUSA i3 MK3S printer, while (**B**) depicts the grid collimator printed with holes arranged in a honeycomb distribution.

The manufactured collimator consists of a block measuring 9.3 × 9.3 × 7.1 cm^3^, which contains 39 conical holes arranged in a hexagonal pattern resembling a honeycomb (Figure 1A,B). Each hole in the grid follows the divergence of the radiation beam with an outer diameter of 0.92 cm and a center-to-center distance of 1.42 cm. The dimensions of the grid allow a projection of the hole at the isocenter (point located 100 cm from the radiation source) with a diameter of 1.42 cm and a center-to-center separation of 2.19 cm, thus providing a maximum radiation field size of 14 × 14 cm^2^. The nominal transmittance of the grid in the blocked areas is (15.6 ± 0.6)%.

Previous studies have reported a degradation in the mechanical properties of PLA-W composites compared to conventional PLA; specifically, a 16–21% decrease in Young’s modulus [25] and a 75–89% reduction in tensile strength [25], as determined by ASTM D638 Type IV standardized tests. Thus, an additional support structure was implemented, which consisted of an outer casing made from PLA doped with carbon fiber, designed to house and stabilize the PLA-W grid. The support structure was fabricated using a 0.15 mm layer height and a 100% infill density with a gyroid internal pattern, providing enhanced mechanical rigidity without compromising printability.

### 2.2. Field Configuration

**Figure 2:**
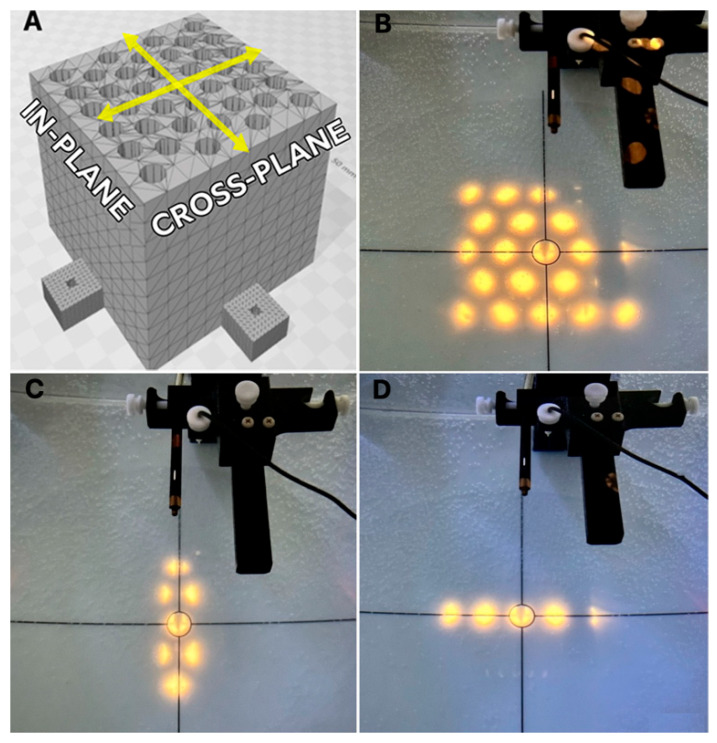
Schematic representation of the different radiation field configurations used for dose profile evaluation. (**A**) Diagram showing the grid geometry with the in-plane (vertical) and cross-plane (horizontal) directions. (**B**) Full beamlet configuration. (**C**) Configuration of central row of beamlets in the in-plane direction. (**D**) Configuration of central row of beamlets in the cross-plane direction.

Different radiation field configurations were generated using the linear accelerator jaws to shape the auxiliary collimation over the grid, with the goal of evaluating dose profiles, i.e., spatial dose distributions perpendicular to the central beam, along the cross-plane and in-plane directions (Figure 2A). The geometries studied included a radiation field produced by the central beamlet alone, a field generated by the central row of beamlets in the in-plane direction (Figure 2C), a field defined by the central row of beamlets in the cross-plane direction (Figure 2D), and a field encompassing all beamlets in the grid (Figure 2B).

To define the central beamlet field, the linear accelerator jaws were set to 2.5 × 2.5 cm^2^. For the in-plane and cross-plane configurations, the jaws were adjusted to 10 × 2.5 cm^2^ and 2.5 × 10 cm^2^, respectively. The full beamlet configuration was shaped using a 10 × 10 cm^2^ collimation, corresponding to the standard reference field size used in dosimetric measurements.

### 2.3. Dosimetric Characterization Protocol

The dosimetric characterization of the grid was performed following the international code of practice TRS-483 [27], considering that each field generated by the collimators at the isocenter corresponds to a small field (1.4 cm diameter). The protocol establishes that the determination of the relative dose $\Omega_{Q_{clin},Q_{10\times 10}}^{\;f_{clin},f_{10\times 10}}$, corresponding to the dose produced by a small field ($f_{small}$) of energy $Q_{small}$ relative to a dosimetric reference field of 10 × 10 cm^2^ ($f_{10\times 10}$) of energy $Q_{10\times 10}$, cannot be directly obtained from the ratios of charges ($M_{Q_{small}}^{f_{small}}/M_{Q_{10\times 10}}^{f_{10\times 10}}$) (corrected by the influence factors $k_{T,P}$, $k_{pol}$, $k_{ion}$) recorded by the measurement system for such field sizes, requiring the introduction of a correction factor $k_{Q_{small},Q_{10\times 10}}^{\;f_{small},f_{10\times 10}}$, specific to the small field size according to the detector used (Equation (1)).

(1) $$\Omega_{Q_{small},Q_{10\times 10}}^{\;f_{small},f_{10\times 10}}=\frac{M_{Q_{small}}^{f_{small}}}{M_{Q_{10\times 10}}^{f_{10\times 10}}}\;k_{Q_{small},Q_{10\times 10}}^{\;f_{small},f_{10\times 10}}$$

However, detectors commonly used for reference field sizes are not suitable for dosimetric measurements in small fields due to their large size compared to the dimensions of the radiation beam. Considering this, different solid-state detectors such as shielded and unshielded silicon diodes, as well as gas detectors like micro-ionization chambers, have been developed with optimized characteristics for the characterization of small fields. Nevertheless, for reference field sizes, the uncertainty of their measurements is higher than those reported with detectors defined as reference for these field sizes (ionization chambers). This is why the TRS-483 [27] protocol introduces the need to complementarily use two types of detectors: one corresponding to a mini-ionization chamber and the other to a small field detector (diode or micro-ionization chamber), along with the introduction of an intermediate field size ($f_{int}$). The intermediate field should have dimensions that are the smallest size allowing measurement by the mini-ionization chamber without being in the small field condition, and for which correction factors are widely studied for the small field detector (reference class detector) for both small and intermediate field sizes (see Equation (2)).

(2) $$\Omega_{Q_{small},Q_{10\times 10}}^{\;f_{small},f_{10\times 10}}={\left[\frac{M_{Q_{small}}^{f_{small}}}{M_{Q_{int}}^{f_{int}}}\;k_{Q_{small},Q_{int}}^{\;f_{small},f_{int}}\right]}_{DET}{\left[\frac{M_{Q_{int}}^{f_{int}}}{M_{Q_{10\times 10}}^{f_{10\times 10}}}\;k_{Q_{int},Q_{10\times 10}}^{\;f_{int},f_{10\times 10}}\right]}_{I.C.}$$

On the other hand, the characterization of the relative depth dose (Percentage Depth Dose: PDD) produced by the grid for different shaped fields presents certain aspects that must be considered depending on the specialized detectors used for small fields. Ionization micro-chambers, with a very small measurement volume (<0.015 cm^3^), exhibit reduced sensitivity. Therefore, during depth measurement scans for short times (typically less than 10 s), leakage signal can become a non-negligible percentage of the dose signal, particularly significant at greater depths or in regions close to the charged particle equilibrium, resulting in increased uncertainties in determining the depth of dose maximum (Z_max_). In the case of diode detectors, unshielded ones exhibit higher sensitivity to low-energy photons from scatter, resulting in an over-response for sizes above 3 cm or with depth. On the other hand, while the metallic shielding in shielded diodes mitigates the effects of scattered photons, it also increases electron fluence, leading to an over-response at shallow depths. For these reasons, PDD measurements of the shaped fields in the manufactured grid were conducted using these three types of detectors to obtain average values that compensate for the effects of each detector.

### 2.4. Detectors

For the determination of the relative reference dosimetry, depth dose curves (PDD), and dose profiles in-plane and cross-plane, three small field point detectors were used: the first two detectors were silicon-diode-type, and the third was a gas-detector-type micro-ionization chamber. The silicon-diode-type detectors included one shielded type from Sun Nuclear, EDGE model (N-type diode) (Sun Nuclear Corporation, Melbourne, Australia) with an active square area of 0.64 mm^2^, and one unshielded type from PTW, SRS 60018 model (P-type diode) (PTW Freiburg GmbH., Freiburg, Germany) with an active circular area of 1.0 mm^2^. The micro-ionization chamber type detector was from Standard Imaging, Exradin A16 model (Standard Imaging, Inc., Middleton, WI, USA) with a sensitive volume of 0.007 cm^3^. For measurements of intermediate field sizes and 10 × 10 cm^2^ (required exclusively for reference dosimetry), a mini-ionization chamber from Standard Imaging, Exradin A1 model (Standard Imaging, Inc., Middleton, WI, USA) with a sensitive volume of 0.057 cm^3^ was used. Data from all detectors were collected by a Sun Nuclear PC Electrometer (Sun Nuclear Corporation, Melbourne, Australia).

### 2.5. Experimental Setup and Measurement Conditions

Measurements were conducted using a Sun Nuclear water phantom, model 1D (Sun Nuclear Corporation, Melbourne, Australia), measuring 30 × 30 × 30 cm^3^, equipped with a robotic arm allowing continuous vertical axis measurements. The distance between the radiation source and the phantom surface was 100 cm, corresponding to 35.1 cm from the outer face of the grid collimator to the surface. The reference measurement depth was 10 cm, resulting in a projected size of the central hole of 1.54 cm (estimated diameter of each beamlet).

For determining the dose profiles (in-plane and cross-plane directions) and output factors of each radiation field with the grid, detectors were aligned relative to the central beamlet generated by the grid. The detector positions were defined as follows: (a) the center of the sensitive volume for the ionization micro-chamber, (b) the position of the semiconductor sensor inside the detector body for both shielded and unshielded diodes (0.57 mm depth from the top surface for the PTW SRS 60018 diode and 0.30 mm for the Sun Nuclear EDGE diode).

For the dose profile measurements, the detectors were moved at 0.1 mm intervals, scanning the positions between −5.0 cm and 5.0 cm in both directions (in-plane and cross-plane). From the acquired data, the Full Width at Half Maximum (FWHM) of the dose peak produced by the central beamlet was determined for the different shaped fields in the grid, in order to define the radiation field size (radiation diameter) produced by the grid holes and how these are modified based on the number and position of generated beamlets. These circular radiation field sizes were converted to equivalent square fields in order to obtain the correction factors $k_{Q_{small},Q_{int}}^{\;f_{small},f_{int}},$ that the TRS-483 protocol associates with each detector.

On the other hand, for the determination of the PDDs of each shaped field, the measurement depth of the A16 ionization chamber was considered at its effective measurement point located 0.72 mm above the center of its sensitive volume, while for the diode detectors, the measurement depth corresponded to their reference position. Measurements were taken over a depth range of 1–20 cm in 1 mm intervals. Based on the collected ionization data, the depth of dose maximum (Z_max_) was determined, and other values were normalized relative to this point in terms of percentages.

Additionally, simultaneous two-dimensional dose distribution measurements were performed using the Sun Nuclear SRS MapCHECK planar diode array and EBT3 radiochromic films. Measurements with the SRS MapCHECK system were conducted both with and without its dedicated phantom, which is specifically designed for stereotactic radiosurgery QA. A sub-region of 7.7 × 7.7 cm^2^ was analyzed, with a spatial resolution that enables simultaneous readout of 5 diodes per 5 × 5 mm^2^ area. EBT3 film measurements were performed within a solid water phantom composed of stacked slabs. In both setups, the source-to-detector distance (SDD) was fixed at 110 cm, resulting in an effective measurement depth of 10 cm for the EBT3 films and 7.62 cm for the SRS MapCHECK system, the latter due to the geometric constraints of its dedicated phantom.

### 2.6. Temporal Evaluation of the Grid

To evaluate the long-term dosimetric stability of the grid, progressive characterization was performed over two years under controlled clinical conditions. The grid was stored and used continuously inside the linear accelerator bunker, where it was exposed to an ambient temperature of 20 ± 2 °C, a relative humidity of (50 ± 10)%, and scattered radiation associated with the equipment’s routine workload. Dosimetric measurements were carried out at different times to evaluate the effects of possible material degradation or geometrical alterations. First, point dose measurements for small fields were performed using the Sun Nuclear Edge diode. One year later, these measurements were repeated using a PTW 60018 diode and an EXRADIN A16 ionization chamber as detectors. Similarly, two-dimensional dose distribution measurements were performed using EBT3 radiochromic films at the time of fabrication and repeated two years later using the SRS MapCHECK system. This methodology enabled the detection of time-dependent dosimetric variations resulting from PLA degradation that alters the internal distribution of tungsten, generating transmittances in lateral regions of the grid volume, as well as inconsistencies in the alignment between the grid structure and the radiation beam.

## 3. Results

### 3.1. Grid Manufacturing

The design and fabrication of the grid were executed with the aim of fitting onto the wedge support of a clinical radiotherapy accelerator, in this case from Varian (Varian Medical Systems, Palo Alto, CA, USA). This meant that the dimensions and total mass of the manufactured piece were constrained to be located at the exit of the head, oriented towards the patient, unlike typical commercial grids which are placed on an additional accessory called a tray holder with the grid facing towards the head. The manufactured grid had a final mass of 3.10 kg and dimensions positioning it at 35.1 cm from the isocenter (the typical treatment reference point).

**Figure 3:**
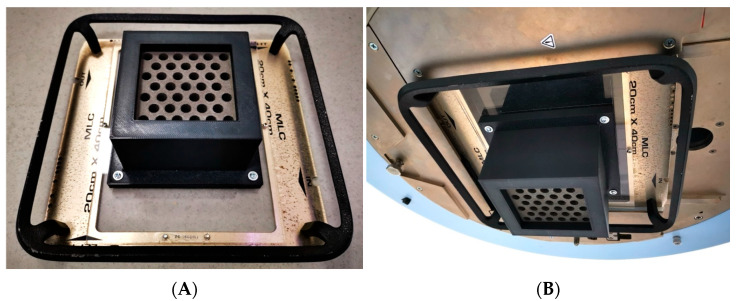
Collimator with PLA support (black color) screwed onto UNIQUE linear accelerator accessory (**A**), and grid collimator inserted into UNIQUE equipment head (**B**).

Although the PLA-W grid exhibits lower mechanical properties compared to conventional PLA, these remain well above the thresholds required to prevent significant structural deformation under expected clinical use conditions. The mechanical loads to which the collimator is subjected—such as its own weight when suspended in various angular orientations and the low rotational speed of the gantry—are well below the material’s critical deformation limits. However, clinical safety regulations require a more secure fixation of the grid to its support. Therefore, a carbon-fiber-reinforced PLA casing, specifically designed to enclose the grid, was ultimately incorporated into the system. This casing was directly fastened to the acrylic base of the wedge accessory used to mount the grid onto the linear accelerator head, as illustrated in Figure 3.

### 3.2. Dose Profiles

The FWHM results are shown in Table 1, where the effective radiation field size for the central beamlet in the single central beamlet configuration was 1.58 ± 0.01 and slightly increased with the number of neighboring beamlets, resulting in 1.59 ± 0.03 for the configuration of central row of beamlets in the cross-plane direction, 1.61 ± 0.01 for the configuration of central row of beamlets in the in-plane direction, and 1.64 ± 0.04 for the full beamlets configuration. It is important to note that theoretically, the diameter of the circular beamlet size calculated at a measurement depth of 10 cm is 1.56 cm considering only the aperture of the central hole, without contribution from scattered radiation by the surrounding holes. As the number of surrounding beamlets contributing dose increases, the field size increases by 1.79%, 3.07%, and 4.99% for the in-plane and cross-plane row of beamlets configuration and full beamlets configuration, respectively.

The evaluation of the change in the ability to modulate radiation over 1 year showed no statistically significant differences, with EDGE diode and EXRADIN A16 ionization chamber measurements showing very high agreement. This would indicate the absence of structural modifications that alter the alignment of the hole collimation.

From the determined FWHMs and their respective conversion to equivalent square field sizes, the correction factors associated with each detector were determined for the subsequent determination of the output factors. The values obtained are summarized in Table 2 for the small field and for the intermediate field.

### 3.3. Output Factor

For the radiation field produced by the single central beamlet configuration, an average output factor of 0.746 ± 0.006 was obtained, while configurations of central row beamlets in the in-plane and cross-plane directions delivered average output factor values of 0.771 ± 0.009 and 0.768 ± 0.009, respectively, corresponding to an increase in output factor of 3.26% and 2.86% compared to the reference value of the single central beamlet configuration. Finally, for the full beamlets configuration, an increase close to 10% was detected with a factor of 0.822 ± 0.008. These results are summarized in Table 3. Additionally, good agreement can be observed between measurements from the detectors, with a difference of less than 2.3% in each measurement between those obtained with the ionization micro-chamber and those made with silicon diode detectors, and statistically indistinguishable discrepancies between the values obtained by the unshielded diode compared to the shielded one.

Once again, the analysis of radiation modulation capability over time revealed no statistically significant differences. Measurements obtained with the EDGE diode and those acquired one year later using the EXRADIN A16 ionization chamber and the PTW 60018 diode showed excellent agreement. This consistency suggests that no structural changes occurred within the grid collimator that might lead to inhomogeneities in the tungsten distribution. Such inhomogeneities would likely alter the grid’s lateral transmittance and be particularly evident in multi-beamlet configurations, potentially resulting in variations in the output factor over time. However, the results confirm that no such variations were observed in the measurements conducted.

### 3.4. Percentage Depth Dose

The depth dose behavior with each of the radiation fields does not show a significant difference, with results falling within the measurement uncertainties, as is shown in Table 4. On average, the maximum dose depth Z_max_ for the four studied radiation field configurations coincides at 1.20 ± 0.20 cm, which is 0.20 cm shallower than the conventional open beam 10 × 10 cm^2^ without the grid.

The analysis of changes in radiation modulation capability over time, specifically by evaluating variations in the depth of maximum dose, once again revealed no statistically significant differences after a one-year interval between measurements. This reinforces the conclusion that no structural alterations occurred within the grid collimator that could lead to inhomogeneities in the tungsten distribution. Such inhomogeneities would be expected to affect the beam quality, potentially resulting in a shift in the depth of maximum dose. However, this effect was not observed in the measurements, further supporting the long-term dosimetric stability of the system.

### 3.5. 2D Dose Distribution and Valley-to-Peak Ratio (VPR)

**Figure 4:**
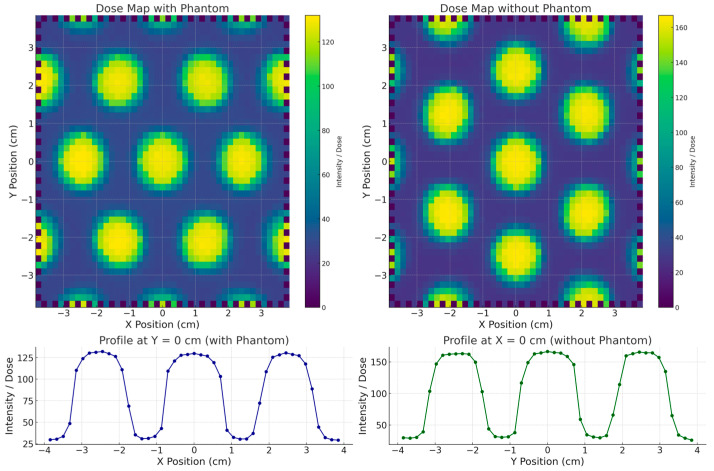
Dosimetric comparison between configurations with and without phantom using SRS MapCheck measurements. Top panels: two-dimensional dose distribution maps of the grid pattern acquired with the phantom in place (**left**) and without phantom (**right**). Bottom panels: lateral dose profile along the *X*-axis at Y = 0 cm for the phantom configuration (**left**), and longitudinal profile along the *Y*-axis at X = 0 cm for the setup without phantom (**right**). Dose values are expressed in relative units.

The surface dose distributions (2D) obtained with and without the stereotactic phantom, along with the corresponding in-plane dose profiles, are presented in Figure 4. The results show that the SRS MapCHECK system yielded the same FWHM value of 1.60 cm for the central beamlet in both measurement configurations. The neighboring beamlets in the in-plane direction showed FWHM values of 1.50 cm and 1.52 cm, respectively—differences that can be attributed to the inherent beam divergence accounted for in the grid design. A similar FWHM value (1.60 cm) was also observed for the central beamlet in the cross-plane direction. The VPR, representing the percentage of transmission through the blocked regions relative to the irradiated beamlet zones, was (17.8 ± 0.5)% in the cross-plane direction and (17.0 ± 0.2)% in the in-plane direction. When the measurement was performed with the stereotactic phantom (equivalent to a water depth of 7.25 cm), the transmission increased to (28.0 ± 0.2)% and (23.5 ± 0.2)% in the cross-plane and in-plane directions, respectively, highlighting the depth-dependent variation in beam transmission.

**Figure 5:**
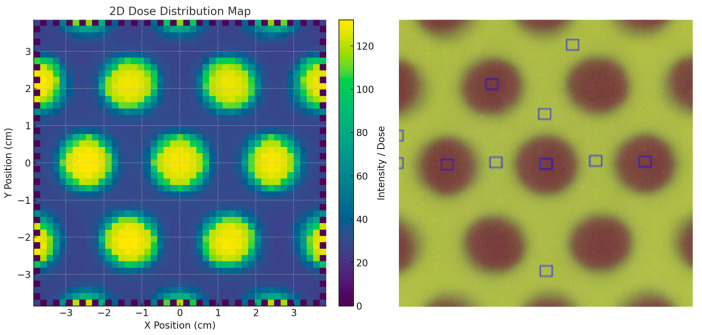
Two-dimensional dose distribution measured with two different systems separated by a two-year interval: (**left**) SRS MapCheck array; (**right**) EBT3 radiochromic film. Blue squares indicate the peak and valley regions used for calculating the valley-to-peak ratio (VPR).

The comparison between the surface dose distribution measured with EBT3 radiochromic film and the SRS MapCHECK is shown in Figure 5. The EBT3 film measurements, conducted at the time of grid fabrication, yielded an FWHM of 1.54 cm and a VPR of 15.8%. It is important to note that EBT3 films were placed directly at the irradiated surface, whereas the SRS MapCHECK detectors (without phantom) are located at an effective depth of 1.0 cm. This difference in measurement depth likely explains the small discrepancy of 3.7% in FWHM and 1.2% in VPR, consistent with the observed increase in transmission at greater depths in the phantom-based measurements.

The comparison of 2D dose distributions acquired two years apart supports the hypothesis of long-term modulation stability in grid structures fabricated using PLA-W under standard environmental conditions (temperature: (20 ± 3) °C; humidity: (50 ± 10)%). The FWHM values of each beamlet within the full beamlet configuration (10 × 10 cm^2^ field) showed only minor variations, attributable to differences between detectors, even with a time gap of two years between measurements. This reinforces the conclusion that no internal deformations occurred that could misalign the collimation holes across the grid surface. Furthermore, the VPR measurements across different field regions remained consistent and within expected ranges, considering the increased depth of measurement with the SRS MapCHECK system. These findings collectively confirm the long-term structural and dosimetric stability of the tungsten distribution throughout the PLA-W grid.

## 4. Discussion

The use of W-PLA composites with very high tungsten concentrations (>90% *w*/*w*) and densities, as previously reported, has proven effective for fabricating spatial radiation modulators with complex geometries and reduced dimensions. These advanced materials preserve the high precision and reproducibility typical of conventional PLA in FFF 3D printing, while enabling sufficient radiation attenuation for clinical applications. However, the correct characterization of these accessories should not only assess the geometric tolerances of the manufactured product, but also the real collimation of the radiation beam in dosimetric terms by evaluating the size of the radiation field and how much it differs from the physical size of the manufactured collimator. In the case of the grid manufactured in this work, each hole was designed with a physical diameter that projects a circular field of 15.6 mm at a distance of 110 cm from the radiation source. The dosimetric measurement of the actual radiation field size is 15.8 ± 0.1 mm, corresponding to a discrepancy of less than 0.13% relative to the physical projection. This allows verification of the real ability to modulate radiation at MV energies by means of this manufacturing technique and the materials used.

An additional effect generated by the presence of multiple simultaneous beamlets is the lateral contribution or superposition of radiation penumbrae that modify the measurement of the individual radiation field of the central beamlet (FWHM). Defects or volumetric inhomogeneities in the manufacturing can cause losses in the spatial modulation of the radiation beams coming from the lateral regions, which would increase the variation of the FWHM of the central beamlet. The measurements show how the FWHM is increased up to 3.8% as a contribution of the adjacent beamlets, but this variation would represent a dosimetric change of less than 1.8% in the expected value for the single central beamlet configuration, reaffirming the radiation modulating capabilities of the material and technique studied. However, as expected, the simultaneous contribution of all the beamlets can increase the total relative dose, reaching increases of up to 10%, offering advantages in certain clinical applications.

Given that SFRT leverages the radiobiological bystander effect while preserving the skin through partial target irradiation, it enables dose escalation without compromising surface integrity. Clinical studies have evaluated various collimator geometries, with the most widely accepted configuration consisting of a 50% open/blocked area ratio [12]. Optimal biological effectiveness has been reported for hole diameters between 1.0 and 1.5 cm and center-to-center spacing between 1.7 and 2.1 cm [30]. Currently, only two commercial grid systems are available—Dot Decimal (Sanford, FL, USA) and Radiation Products Design (Albertville, MN, USA)—both featuring 1.4 cm hole diameters and 2.1 cm spacing, projected at 100 cm source-to-axis distance (SAD). This standard geometry was adopted in the present study to allow a direct comparison of materials (PLA-W vs. brass and Cerrobend) and manufacturing techniques (3D printing vs. traditional subtractive manufacturing).

The observed FWHM variations (<3.8%) fall within clinically accepted geometrical tolerances and have minimal impact on the intended radiobiological effect, influencing only minor adjustments in small-field dosimetry. Given that SFRT is typically delivered in a single or dual-field fraction, these variations are not expected to significantly affect dose-volume histograms (DVHs).

Although the literature has reported possible alterations in the mechanical properties of PLA associated with radiation exposure and environmental aging, the resulting structural degradation is generally negligible. In fact, even under extreme irradiation scenarios, equivalent to approximately 25,000 conventional treatment fractions (approx. 30 kGy), no relevant damage to the polymer matrix has been observed [31]. This threshold is well above the level of scattered and indirect radiation that a PLA-W grid collimator would receive in a clinical setting over its lifetime. Consequently, the results support the mechanical and dosimetric stability of the collimator over time under realistic clinical use conditions.

Finally, when comparing the performance of the manufactured grid with the commercially available alternatives [12,32,33,34], a high level of agreement is achieved for the main clinically relevant characteristics, which are summarized in Table 5.

## 5. Conclusions

The introduction of new metal–PLA composite filaments for 3D printing based on metals with high atomic number and high density (such as tungsten) opens new scenarios in the ability to design complex and specialized parts and accessories for clinical environments, especially in applications involving radiation. These compounds have the potential to replace highly toxic elements which are not allowed by current clinical guidelines to be included in the composition of clinical equipment and accessories. In particular, the main results obtained with the manufactured grid include the following:

- Deviations between generated radiation fields and generated physical fields of less than 0.13%.
- Variation in the field size of the central beamlet due to lateral transmission of the beam when generating adjacent beamlets, less than 3.8%.
- Similar maximum depth doses for all configurations studied (1.20 ± 0.20 cm).

## Figures and Tables

**Table 1:** FWHM of the central beamlet for different radiation field configurations, measured using the SNC EDGE detector (6 months post-manufacture) and the EXRADIN A16 and PTW 60018 detectors (18 months post-manufacture).

Detector	Radiation Field Configuration			
	Single Central Beamlet	Row of Beamlets in the In-Plane Direction	Row of Beamlets in the Cross-Plane Direction	Full Beamlets
EXRADIN A16	1.58 cm	1.60 cm	1.58 cm	1.62 cm
PTW 60018	1.59 cm	1.65 cm	1.63 cm	1.68 cm
SNC EDGE	1.57 cm	1.57 cm	1.57 cm	1.61 cm
**Average**	**1.58 ± 0.01 cm**	**1.61 ± 0.04 cm**	**1.59 ± 0.03 cm**	**1.64 ± 0.04 cm**

**Table 2:** Correction factors obtained for diode SNC EDGE, ionization chamber EXRADIN A16 and diode PTW 60018, for the small and intermediate field.

Detector	Single Central Beamlet	Row of Beamlets in the In-Plane Direction	Row of Beamlets in the Cross-Plane Direction	Full Beamlets	Open Field 3 × 3 cm^2^
EXRADIN A16	1.011	1.010	1.010	1.010	1.000
PTW 60018	0.996	0.997	0.996	0.998	1.011
SNC EDGE	0.982	0.982	0.982	0.984	0.999

**Table 3:** Output factors obtained for different radiation field configurations, measured using the SNC EDGE detector (6 months post-manufacture) and the EXRADIN A16 and PTW 60018 detectors (18 months post-manufacture).

Detector	Output Factor			
	Single Central Beamlet	Row of Beamlets in the In-Plane Direction	Row of Beamlets in the Cross-Plane Direction	Full Beamlets
EXRADIN A16	0.75	0.78	0.78	0.83
PTW 60018	0.74	0.77	0.76	0.82
SNC EDGE	0.74	0.77	0.76	0.82
**Average**	**0.75 ± 0.01**	**0.77 ± 0.01**	**0.77 ± 0.01**	**0.82 ± 0.01**
**Increase**	**---**	**3.26%**	**2.86%**	**10.09%**

**Table 4:** Maximum dose depth values obtained for different radiation field configurations, measured using the SNC EDGE detector (6 months post-manufacture) and the EXRADIN A16 and PTW 60018 detectors (18 months post-manufacture).

Detector	Z_máx_ (cm)			
	Single Central Beamlet	Row of Beamlets in the In-Plane Direction	Row of Beamlets in the Cross-Plane Direction	Full Beamlets
EXRADIN A16	1.20 ± 0.30	1.20 ± 0.30	1.10 ± 0.30	1.10 ± 0.30
PTW 60018	1.30 ± 0.20	1.20 ± 0.20	1.30 ± 0.20	1.30 ± 0.20
SNC EDGE	1.10 ± 0.20	---	---	1.20 ± 0.20
**Average**	**1.20 ± 0.20**	**1.20 ± 0.20**	**1.20 ± 0.20**	**1.20 ± 0.20**

**Table 5:** Dosimetric comparison between the SFRT grid manufactured with PLA-W via 3D printing (this study) and commercial grid parameters reported in the literature [12,32,33,34].

Dosimetric Parameter	PLA-W Grid (This Study)	Commercial Grids (Literature)
Mean FWHM (central beamlet only)	1.58 ± 0.01 cm	1.4–1.6 cm
Output factor (central beamlet only)	0.746 ± 0.006	0.74–0.76
Output factor (full beamlets)	0.822 ± 0.008	0.81–0.83
Mean FWHM variation (full beamlets)	≤3.8%	~3–5%
Valley-to-peak ratio	17.0–17.8%	10–20%
Depth of maximum dose	1.20 ± 0.20 cm	1.2–1.3 cm (6 MV)

## Data Availability

Data are contained within the article.
